# Efficacy and Safety of an Ophthalmic DMPC-Based Nanoemulsion in Patients with Dry Eye Disease: A Phase I/II Randomized Clinical Trial

**DOI:** 10.1155/2023/1431473

**Published:** 2023-04-10

**Authors:** Leopoldo M. Baiza-Durán, Patricia Muñoz-Villegas, Alejandra Sánchez-Ríos, Oscar Olvera-Montaño

**Affiliations:** Regional Medical Affairs Department, Laboratorios Sophia SA de CV, Guadalajara, Mexico

## Abstract

**Purpose:**

The goals of this study were to evaluate the safety and efficacy of an ophthalmic 1,2-dimyristoyl-sn-glycero-3-phosphocholine (DMPC)-based nanoemulsion (Nanodrop®) in patients with dry eye disease (DED).

**Methods:**

This was a randomized phase I/II multicentric, prospective, double-blind clinical trial. Patients (phase I: *n* = 25 and phase II: *n* = 101) were assigned to receive either PRO-176 (Nanodrop®) or Systane Balance® (control) for 29 days. Once the visits of the first 25 subjects were completed, if there were less than 20% of unexpected adverse events (AEs), related to PRO-176, recruitment was continued until the sample was completed for noninferiority (efficacy) analysis (phase II, *n* = 126). Efficacy endpoints were the ocular surface disease index (OSDI), tear break-up time (TBUT), epithelial defects, best corrected visual acuity (BCVA), and the incidence of expected AE.

**Results:**

For the phase I portion of the study, there were no differences between groups regarding the incidence of AE. All related-AE symptoms in both groups were mild and expected. For the phase II subset, there was a significant reduction in OSDI scores at day 29 and noninferiority between treatments was confirmed (*p*=0.650, CI 95% [−8.7, 5.5]). Similar improvement was observed for TBUT although no significant intergroup differences were found (*p*=0.518, CI 95% [−0.08, 1.6]). There were no significant differences between treatments for epithelial staining or safety parameters.

**Conclusions:**

Topical application of PRO-176 is as safe and effective as the controls. Both groups were clinically similar in terms of efficacy and safety. The results support the hypothesis that ophthalmic DMPC-based nanoemulsion may improve clinical parameters and symptoms in patients with DED. This trial is registered with NCT04111965.

## 1. Introduction

Dry eye disease (DED) is a common and complex pathology of the ocular surface that affects 6–34% of the word's adult population and significantly reduces quality of life [1–3].

This disorder is caused by an insufficient or inefficient and unstable tear film that causes a range of signs and symptoms, including discomfort, foreign body sensation, hyperemia, ocular pain, visual alterations, and disruption of the outermost layer of the cornea detected by a fluorescein eye stain test, reduced tear film break-up time (TBUT), and so on [4, 5].

The described alterations of the tear film may be caused by decreased production of the tear's elements and/or due to its elevated evaporation rate. This form of DED is in many cases caused by disruptions of the lipidic component of the tear [5, 6]. The lipid layer is composed of fatty acids and fatty alcohols, as well as lipids, mainly the nonpolar type. However, small amounts of phospholipids or polar lipids are also present, and their hydrophilic properties act as an interface between the lipid and aqueous layers [7, 8].

Even though the biochemical profile, rheological and clinical properties of the tear film's lipid layer are not completely understood yet, it is known that one of its main purposes is to decrease the evaporation rate of the tear film [5–7]. In patients with deficiency of this portion of the tear, like in meibomian gland disfunction (MGD), one of the treatment goals must be tacking the lipid insufficiency to treat the associated dry eye. Emollient-containing eye drops are useful in these cases, aiding in the replenishment of lipid-deficient tear films and therefore reducing tear evaporation and contributing to tear film stability [5]. However, some of the adverse effects of these emollient eyedrops include temporary blurry vision and reduced contrast sensitivity due to their viscous consistency [5].

A nanoemulsion contains small particles (10–1000 nm) and both lipidic and aqueous components. Formulating a nanoemulsion eye drop allows an aqueous-like dispersion over the ocular surface, adding lipids to the tear film and therefore avoiding the emollient-related adverse events.

The rheological properties of propylene glycol, a synthetic lubricant used in several commercially available eyedrops (some of which include lipids in their formulations), modify the tear film's viscosity while the lipidic phase of the nanoemulsion acts as a surfactant, aiding in the adequate dissemination of nonpolar lipids in the aqueous component of the tears. The resulting barrier between the aqueous and lipidic phases supports the nonpolar phase, decreasing the evaporation rate of the tear film [9].

Phase I/II clinical trials test the safety, side effects, and how well a certain type of disease responds to a new treatment [10]. In the present study, safety and efficacy of the ophthalmic nanoemulsion composed of a novel oily phase of castor oil and 1,2-dimyristoyl-sn-glycero-3-phosphocholine, consequently called DMPC-based nanoemulsion (PRO-176), were evaluated in terms of reduction of signs and symptoms, thus helping in the treatment of DED. It was compared with a commercially available and widely distributed emulsion (Systane Balance®) indicated for temporary relief of some of the symptoms associated to DED. This reference product was selected since its formulation is similar to PRO-176 and its safety and efficacy have been previously established.

## 2. Materials and Methods

### 2.1. Study Design

This was a multicenter, comparative, prospective, parallel group, phase I/II clinical trial. The study was registered in clinicaltrials.gov as NCT04111965. It was conducted in five centers in Mexico. An ethics committee in each center reviewed and approved the study's protocol and informed consent (see Acknowledgments section). The research was conducted in compliance with the Declaration of Helsinki and in accordance with Good Clinical Practices Standards. All patients who participated in this study provided written and signed informed consent. Patients in phase I were recruited between December 1, 2020 (FPFV) and March 16, 2021 (LPLV). Patients in phase II were recruited between June 7, 2021 (FPFV) and December 30, 2021 (LPLV). This study's design and methods are based on previously published clinical trials [11, 12].

This study consisted of two phases: phase I and phase II. Phase I aimed to confirm the safety of PRO-176 in patients with DED. Phase II aimed to investigate efficacy with the primary endpoint of OSDI score, taking place only after it was confirmed that a less than 20% of unexpected AE related to PRO-176 were present during phase I, and therefore allowing recruitment to continue until the sample was completed for efficacy analysis.

### 2.2. Participants

Inclusion criteria comprehended patients either men or women (aged ≥18 years), with DED diagnosis (OSDI score ≥13, plus one of the following: corneal staining with more than 5 sites, conjunctival staining with more than 9 sites, or TBUT <10 seconds). Exclusion criteria were the use of topical ocular drops and systemic medication that may have affected the study's results (systemic steroids, immunomodulators, and tetracyclines), patients with conjunctivitis, anterior blepharitis, demodex, eye parasitic infections, unresolved eye trauma, healing disorders of the ocular surface, and presence of any illness that could interfere with study parameters (e.g., glaucoma or retinal diseases), history of penetrating keratoplasty, present a distance best corrected visual acuity (BCVA) of 20/200 or worse in one of the eyes, use of contact lenses, previous history of any ophthalmic surgical procedure within 3 months before baseline, subjects with a single functional eye, and patients who were pregnant or at risk for pregnancy (having no birth control treatment) or breastfeeding.

### 2.3. Treatment and Evaluations

A total of one hundred and twenty-six subjects (63 subjects per group) were randomized 1 : 1 to receive a control ophthalmic emulsion of propylene glycol 0.6% (Systane Balance®, Alcon Laboratories, Inc., Fort Worth, TX, USA (phase I, *n* = 12 and phase II, *n* = 51)) or PRO-176 ophthalmic nanoemulsion (Nanodrop®, Laboratorios Sophia, SA de CV, Zapopan, Jalisco, Mexico; (phase I, *n* = 13 and phase II, *n* = 50)). Subjects' selection and evaluation bias was minimized by random assignment to treatment groups and by treatments-assignment masking. Randomization numbers were generated using computer software (SAS Institute, Inc., Cary, NC, USA). Subjects instilled a drop of study drug topically in the inferior conjunctival sac of both eyes four times per day (QID) for 29 days. If patients required administering additional drops, they could do it as needed (Pro re nata schedule, PRN) [12]. All the researchers and other sponsoring team members were blind to treatment assignment throughout the study. Follow-up visits took place on days 8 and 29 after randomization (day 1). A safety call was carried out one week after the final visit (35^th^ ± 1 day).

The study drug was discontinued if either the principal investigator or patient judged that it was not in the latter's best interest to continue or if a female patient became pregnant.

### 2.4. Phase I: Safety Assessment

**Figure 1:**
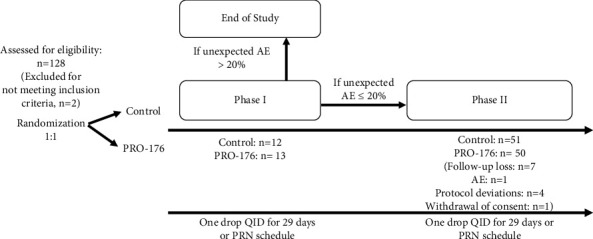
Clinical study design. Phase I was a safety cohort and phase II was an expanded cohort for efficacy assessment.

Because this is the first trial of PRO-176 in the target population, a safety analysis (20% of the total sample, 25/126) was conducted as the phase I subset of the study. Safety was measured by the incidence of unexpected AE related to PRO-176. The expected AE (listed versus unexpected), their causality and relatedness assessment (related, possibly related, and not related), and their severity (mild, moderate, and severe) were analyzed [12–16]. The following information about each AE was collected: severity, event duration, frequency, seriousness, relationship to investigational product (PRO-176 and control), action taken, and outcomes. For AE evaluation, any method used in the trial to elicit subject/reported AE, such as a diary, checklist, and memory aid, whether applied face-to-face or otherwise was considered. All AE occurring during the clinical protocol were recorded and included in the analysis. When the visits of the first 25 subjects were completed (safety cohort), if there were less than 20% of unexpected AE related to PRO-176, recruitment was continued until the sample was completed for efficacy analysis (phase II, efficacy cohort) (see Figure 1). After obtaining clearance through the PRO-176 safety data analysis, research centers continued the trial, and the phase II section was conducted.

### 2.5. Phase II: Efficacy Assessment

Phase II was an expanded efficacy cohort. The primary efficacy endpoint was demonstrating the noninferiority of PRO-176 compared to the control emulsion regarding efficacy as treatment of DED by means of the OSDI score, over the 29-day treatment period [17]. Noninferiority was determined as a difference inferior to 5 points in the OSDI test score between PRO-176 and control. Secondary outcomes were TBUT (number of seconds) performed at each follow-up visit, presence of the epithelial defects evidenced by fluorescein corneal and lissamine green conjunctival staining tests (FCS and LGCS, respectively), the BCVA evaluated through the Snellen chart (to provide standardized and well-controlled assessments of visual acuity during the study, the same lighting conditions were used during the entire study visits), and the incidence of expected AE. Surface dye staining was classified with a scale from 0 to 6, in accordance with the percentage of the affected area (using the SICCA scale, where 0 = no staining) [18].

Finally, since measurements obtained from the right and left eyes are usually correlated [19], data from the right eye were used to give a single data point per subject [20, 21].

### 2.6. Statistical Analysis

Statistical analyses were carried out using the R statistical software package (The R Foundation for Statistical Computing; https://www.R-project.org). All data are expressed as mean ± SD unless indicated otherwise. Sample size calculation was performed to test the reduction in OSDI score after one month of treatment, with an expected difference of at least 5 points with an alpha of 0.05 and a power of 80% [22, 23]. Therefore, 101 subjects were considered per group, allowing as much as 25% of excluded cases in the event of major protocol deviations. A total of 126 subjects were randomized. Statistical evaluations for differences were performed with the Student's *t*-test for continuous data. The 95% confidence interval (CI) of these differences was computed. The ordinal variables were analyzed using *p* × *q* contingency tables, and the differences were calculated with the Pearson chi-square test. All statistical analyses performed in this study were with a *p* < 0.05.

## 3. Results

### 3.1. Characteristics of the Participants

This study enrolled 126 subjects (ITT population, from both phases), from which 11 discontinued their participation due to presentation of an AE (1/11, 9.1%), patient's decision unrelated to AE (1/11, 9.1%), follow-up loss (6/11, 54.5%), or protocol deviations (3/11, 27.3%). Two patients were excluded from the PP analysis population (effectiveness evaluation) due to poor compliance, determined as adherence <70% to the indicated treatment (see Figure 1).

Demographic and baseline characteristics were similar between treatment groups, showing no significant differences (see Table 1). The mean age ± standard deviation (SD) was 46.2 ± 16.5 years (range 62 years), and 69% of subjects were female. All subjects in each group were diagnosed with DED (see Materials and Methods section). Drug administration was similar between groups, and the mean number of applications during the 8 first days of the study was 4.26 ± 1.2 drops per day for control group vs. 4.11 ± 0.7 drops per day for PRO-176 (*p*=0.414). At day 29, the QID scheme continued in both groups (4.20 ± 0.9 drops per day vs. 4.14 ± 0.8 drops per day, respectively, *p*=0.722). Only 4 patients in each group followed a PRN schedule (≥5 drops per day).

### 3.2. Phase I: Safety by Unexpected-Related Adverse Events (AEs)

Safety data were analyzed for the first 25 subjects who completed their follow-up visits up to day 35 (safety call). The mean age ± SD for the control group was 38.7 ± 11.3 years vs. 45.1 ± 16.2 years for PRO-176 (*p*=0.369). For the control group, 58.3% of subjects were female vs. 69.2% for PRO-176 (*p*=0.688). A total of 34 AEs were reported by 64% (16/25) of the subjects randomized during the phase I period of the study. There were no significant differences between treatments for the incidence of AE (*p*=0.688). In each group, 17 AE were reported, all of them classified as mild. Regarding the expectedness of AE, 32.4% were not treatment-related (11/34), and 67.6% were related (control 84.4% vs. 52.9% for PRO-176, *p*=0.141). All related-AE in both groups were expected. During the phase I period, the most common class of reported AE was burning (29.4%), followed by itching (11.8%), and ocular pain, as well as foreign body sensations in the same proportion (8.8%).

Finally, causality assessment means finding a causal association or relationship between a treatment and reaction. There are multiple criteria or algorithms available for stablishing a causal relationship in case of adverse drug reaction. Based on the World Health Organization-Uppsala Monitoring Center scale, 35.3% of AE were possible, 47.1% probable or likely, and 17.6% unlikely. There were no differences between groups (*p*=0.246) (see Table 2).

### 3.3. Phase II: Efficacy by Ocular Surface Index (OSDI)

**Figure 2:**
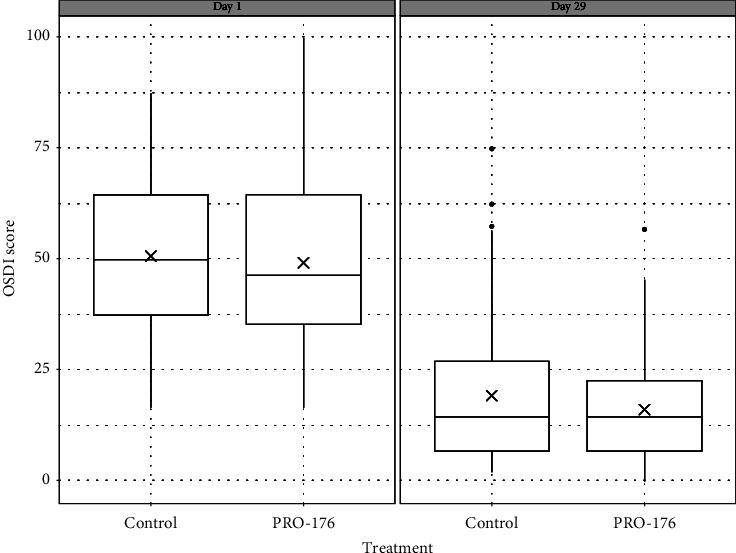
Phase II: OSDI score, change from baseline in the PP population (*n* = 113). The decrease in both groups was statistically significant at 29 days (*p* < 0.0001). However, there was no significant difference in the OSDI between groups. The cross indicates the mean.

In the PP population analysis (*n* = 113), there was a significant reduction in OSDI score after 29 days of treatment; this analysis showed that both groups had a similar score reduction (i.e., no significant differences were found). Noninferiority between treatments was confirmed. The mean change ± SD from baseline to day 29 was −31.5 ± 19.3 for control and −33.1 ± 18.6 for PRO-176; no significant between-group differences were observed (*p*=0.650, CI 95% [−8.7 and 5.5]) (see Table 3). The final OSDI score was 19.3 ± 16.7 for control vs. 16.2 ± 12.3 for PRO-176 (*p*=0.130). At day 29, a reduction in OSDI score ≥30% (versus the baseline score) was observed in 90.3% of the subjects (88.1 vs. 92.6%), ≥50% in 74.3% (72.9% vs. 75.9%) and a reduction ≥70% in 46%, (50.8% vs. 40.7%). No significant differences were observed between groups (*p* values: 0.317, 0.439, and 0.187, respectively) (see Figure 2). The OSDI analysis identified 6 subjects assigned to control group who did not present at least 5 points of improvement in their OSDI score at 29 days (age 50 ± 18.7 years, 83.3% female).

### 3.4. Tear Break-Up Time (TBUT)

**Figure 3:**
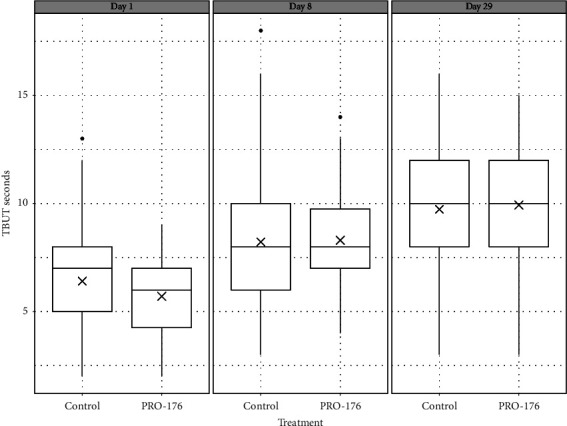
Phase II: TBUT with fluorescein in seconds, change from baseline in the PP population (*n* = 113). The increase in all groups was statistically significant after 8 and 29 days (*p*=0.0001). Though there was no significant difference in the TBUT between groups. The cross indicates the mean.

After 29 days of treatment, the TBUT showed significant improvement compared to day 8 in both the control group (*p*=0.009, CI 95% [−2.7, −0.4]) and the PRO-176 group (*p*=0.003, CI 95% [−2.7, −0.58]), without differences between them (*p*=0.630, CI 95% [−0.8, 1.3]). After 8 days of treatment, the mean change ± SD was 2.02 ± 2.9 seconds for the control group vs. 2.28 ± 2.9 seconds for PRO-176, no significant differences between treatments were found (*p*=0.630, CI 95% [−0.8, 1.3]). By day 29, the control group had a mean change of 3.56 ± 3.4 seconds vs. 3.94 ± 2.8 seconds for PRO-176, showing no statistically significant difference between groups (*p*=0.518, CI 95% [−0.08, 1.6]) (see Table 3). There was no significant difference between groups for the TBUT at any time points (*p* values: 0.079, 0.877, and 0.727, for days 1, 8, and 29, respectively) (see Figure 3).

### 3.5. Fluorescein Corneal Staining and Lissamine Green Conjunctival Staining

A decrement in the score for FCS at day 8 was evidenced, with the control group having a change of −0.37 ± 0.98 vs. −0.28 ± 0.79 for PRO-176 group (*p*=0.573, CI 95% [−0.23, 0.42]). Meanwhile, the improvement in the FCS score at the final visit was −0.49 ± 1.1 for control vs. −0.39 ± 1.0 for PRO-176. No significant differences between treatments were observed (*p*=0.615, CI 95% [−0.30, 0.51]) (see Table 3).

There was a narrowed decrease in LGCS score since it was similar from baseline, at day 8 (−0.32 ± 0.86 vs. −0.11 ± 0.634, *p*=0.139, CI 95% [−0.07, 0.49]) and day 29 (−0.37 ± 0.81 vs. −0.39 ± 0.86, *p*=0.919, CI 95% [−0.32, 0.29]) for both control and PRO-176 groups. There was no significant difference between groups for change from baseline at any time point (*p* > 0.05) (see Table 3).

### 3.6. Best Corrected Visual Acuity (BCVA)

After the intervention, the BCVA (decimal) did not change significantly from baseline to final visit in neither group (0.019 ± 0.13 vs. 0.022 ± 0.08, *p*=0.863, and CI 95% [−0.04, 0.04]). There were no significant differences for the mean value of BCVA between treatments at baseline (*p*=0.764, CI 95% [−0.08, 0.06]), day 8 (*p*=0.510, CI 95% [−0.04, 0.09]), nor at day 29 (*p*=0.985, CI 95% [−0.06, 0.06]) (see Table 3).

### 3.7. Expected Adverse Events

In the ITT population (*n* = 126), of the total AE reported in both phases, 20.4% related-AEs were unexpected for PRO-176 vs. 17.5% for the control group (*p*=0.198). Of them, 95.7% were mild and 4.3% moderate, without differences between groups (*p*=0.635). According to their causality assessment, 8.7% of AE were possible, 6.5% were probable or likely, and 84.8% were unlikely, without differences between groups (*p*=0.230). For PRO-176, 4.9% of unexpected AE were related to the drug vs. 1% in control group.

**Figure 4:**
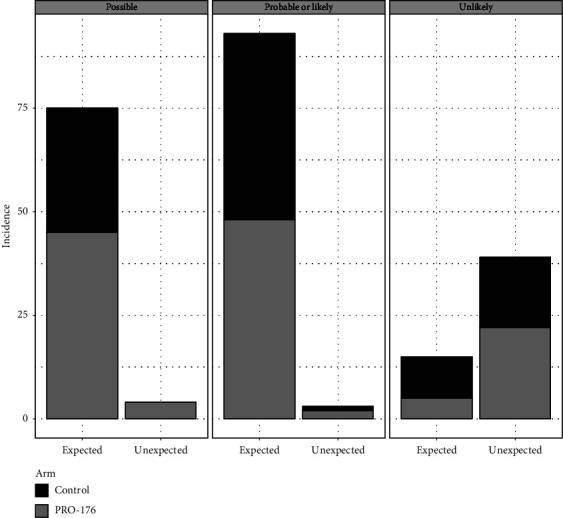
Treatment-related adverse events in ITT population (*n* = 126). Causality assessment versus expectedness. At the end of phase II, for control group at least 1% of total unexpected AE were related to treatment vs. 4.9% for PRO-176. No statistically significant differences were found between control and PRO-176, *p* > 0.05 in all comparisons.

A total of 79.6% of the reported AEs were expected (e.g., Investigator's Brochure for PRO-176 and/or package insert/summary of product characteristics for the approved/control product) and 77% related-AE for PRO-176 vs. 82.5% for control (*p*=0.198). 40.8% were classified as possible (PRO-176: 45.7% vs. 35.3% for control), 51.4% as probable or likely (50% vs. 52.9%, respectively), and 7.8% as unlikely (4.3% vs. 11.8%). No significant differences between groups were observed (*p*=0.106). The treatment-related AE, based on their causality assessment and expectedness, is shown in Figure 4. There was a total of 95% mild AE (98.9% in PRO-176 and 90.6% in control group), 3.9% moderate AE (only in control group), and 1.1% serious AE (one in each group). The most common class of reported AE was burning (26.6%), followed by itching (11.4%) and blurry vision (6.6%), without differences between groups (*p*=0.089).

## 4. Discussion

Dry eye disease (DED) is a common and complex disorder caused by an insufficient or inefficient and unstable tear film. It causes a range of signs and symptoms which alter the quality of life of those who from suffer it [5].

One of the elements of the tear film is the lipid layer, which is comprised by elements such as fatty acids, fatty alcohols, and polar and nonpolar lipids. It is well known that this layer has the ability to decrease the tear film's evaporation rate [5–8].

For patients who suffer conditions that alter the lipid layer, such as MGD, emollient-containing eye drops are believed to replenish their lipid-deficient tear films, and therefore reduce tear evaporation and contribute to tear film stability [5].

PRO-176 is a nanoemulsion that supplements lipids to the tear film while maintaining aqueous-like dispersion over the ocular surface, both because of the small particles it contains and through the incorporation of propylene glycol in its formulation [9].

This study evaluated the safety (phase I) and efficacy (phase II) of PRO-176, in comparison to the commercially available Systane Balance®. Following a complete pipeline of development, PRO-176 had already been subjected to a cytotoxicity preclinical evaluation in NHEK-Neo cells with satisfactory results [9]. On the other hand, during clinical evaluations, safety was measured during the phase I portion of this trial through the incidence of unexpected AE-related to the product. During this period, a total of 34 adverse events were reported (17 for each group), all of them classified as mild. A total of 67.6% were considered related to the product, and all of them were expected. The most common AEs were burning, itching, ocular pain, and foreign body sensation. There were no differences between groups neither for the incidence of AEs, nor for the causality assessment.

After safety evaluation during the phase I segment of the study, after an incidence of less than 20% nonexpected AE was confirmed, recruitment continued for the phase II stage of the study. For efficacy, during the phase II portion of the trial, the primary endpoint was the OSDI score obtained over the treatment period, compared to that of the previously described reference product. The mean change in the OSDI score from baseline to day 29 was −31.5 ± 19.3 for the reference product and −33.1 ± 18.6 for PRO-176, with no significant differences between groups. Reduction in the OSDI score results ≥30% was observed in 90.3% of the subjects, and ≥50% in 74.3%. Once again, no significant differences were found between groups asserting the noninferiority of PRO-176 compared to the reference product.

Secondary variables included TBUT, corneal fluorescein staining, conjunctival lissamine green staining, BCVA, and incidence of expected AEs. There were no significant differences between groups for any of these evaluations, demonstrating the similar efficacy and safety profiles of both products. Furthermore, QID dosing of PRO-176 and the reference product was not different to PRN dosing between treatments and in terms of the endpoints evaluated. This data correlates to previous findings, which proposed that regular use of artificial tears might provide better symptomatic relief that PRN use [12].

DED is a condition that affects millions of lives around the world, and even though the understanding of the complex interactions of the tear film, its components, and the ocular surface grows continuously, there is still continuously growing research regarding its diagnosis, classification, and treatment.

Lipid insufficiency in the tear film has been identified as one of the causes of its increased rate of evaporation, and it is now clear that most cases of DED are not limited to one type of etiology but rather to a mixture of both aqueous-deficient and evaporative DED [24]. Therefore, it is necessary to develop an ocular lubricant with the ability to restore the lipid requirements of the tear film and prevent evaporation, while sparing its users from the adverse events related to emulsions when applied on the ocular surface. PRO-176, as a nanoemulsion, fits this profile.

This study's limitations included the lack of classification of the type of DED diagnosed in the subjects. It is possible to assume that if only subjects with a certain diagnosis of evaporative or mixed DED patients were recluted, the results could have been more conclusive [24, 25]. The fact that no specialized tools were used to measure the lipid layer could also be considered a limitation [6, 26, 27].

## 5. Conclusion

In this study, the safety and efficacy of PRO-176 were proven by significant reduction in symptoms related to DED reported by the users and exposed by the OSDI score evaluation. The results support that ophthalmic DMPC-based nanoemulsion (PRO-176) may improve clinical parameters and symptom in patients with DED. Furthermore, it presented no significant differences with an already known and commercially available eye drop, thus offering an alternative for the treatment of patients with evaporative or mixed DED.

## Figures and Tables

**Table 1:** Initial characteristics of each group (*n* = 63 subjects per group, from both phases).

Variable	Control	PRO-176	*p* values
Male/female^1^, %	36.5/63.5	25.4/74.6	0.247
Age^2^, years ± SD	44.6 ± 15.4	47.8 ± 17.4	0.269
Comorbidities^1^, *n* (%)	21 (33.3)	23 (36.5)	0.852
OSDI^2^, score ± SD	50.3 ± 18.3	48.9 ± 19.2	0.691
TBUT^2^, seconds ± SD	6.4 ± 2.2	5.9 ± 2.0	0.261
FCS^2^, score ± SD	0.97 ± 1.1	0.98 ± 1.0	0.934
LGCS^2^, score ± SD	0.84 ± 0.8	0.92 ± 0.7	0.552
BCVA^2^, decimal ± SD	0.89 ± 0.2	0.88 ± 0.2	0.697

*Notes*. ^1^Chi-square test, ^2^Student's *t*-test. All *p* values >0.05 between groups. Abbreviations. FCS, fluorescein corneal staining; BCVA, best corrected visual acuity; LGCS, lissamine green conjunctival staining; OSDI, ocular surface disease index; SD, standard deviation; TBUT, tear break-up time.

**Table 2:** Phase I: treatment-related adverse events (34 AE/25 subjects).

	Control (*n* = 12)	PRO-176 (*n* = 13)
Patients with AE, *n* (%)	7 (58.3)	9 (69.2)
Related-AE, *n* (%)	14 (82.4)	9 (52.9)
Unexpected/expected related-AE, %	0/100	0/100
Unrelated-AE, *n* (%)	3 (17.6)	8 (47.1)
Unexpected/expected unrelated-AE, %	33.3/66.7	50/50
Causality term, *n* (%)		
Possible	6 (35.3)	6 (35.3)
Probable or likely	9 (52.9)	7 (41.2)
Unlikely	2 (11.8)	4 (23.5)
Nonocular AE, *n* (%)	0 (0)	1 (5.9)
Ocular AE, *n* (%)	17 (100)	16 (94.1)
Burning eye, %	23.5	35.3
Itching	23.5	0
Ocular pain, %	11.8	5.9
Foreign body sensation, %	5.9	11.8
Other, %	35.3	47
Total AE, *n*	17	17

*Notes*. Data show frequency (percentage), *n* = 25 randomized subjects. No significant differences between groups, all *p* values (the chi-square) were >0.05. For both groups, 100% of related-adverse events were expected. Abbreviations. AE, adverse event.

**Table 3:** Phase II: change from baseline (day 1) at 8 and 29 days follow-up.

	Control (*n* = 59)	PRO-176 (*n* = 54)	*p* values [CI 95%]
OSDI, score ± SD			
Study day 29	−31.5 ± 19.3	−33.1 ± 18.6	0.650 [−8.7, 5.5]
TBUT, seconds ± SD			
Study day 8	2.02 ± 2.9	2.28 ± 2.9	0.630 [−0.8, 1.3]
Study day 29	3.56 ± 3.4	3.94 ± 2.8	0.518 [−0.08, 1.6]
FCS, score ± SD			
Study day 8	−0.37 ± 0.98	−0.28 ± 0.79	0.573 [−0.23, 0.42]
Study day 29	−0.49 ± 1.1	−0.39 ± 1.0	0.615 [−0.30, 0.51]
LGCS, score ± SD			
Study day 8	−0.32 ± 0.86	−0.11 ± 0.63	0.139 [−0.07, 0.49]
Study day 29	−0.37 ± 0.81	−0.39 ± 0.86	0.919 [−0.32, 0.29]
BCVA, decimal ± SD			
Study day 8	−0.007 ± 0.14	0.013 ± 0.18	0.488 [−0.04, 0.08]
Study day 29	0.019 ± 0.13	0.022 ± 0.08	0.863 [−0.04, 0.04]

*Notes*. Data from PP population (*n* = 113). The Student's *t*-test, all *p* values >0.05 between groups. Abbreviations. CI 95%, 95% confidence interval; BCVA, best corrected visual acuity; FCS, fluorescein corneal staining; LGCS, lissamine green conjunctival staining; OSDI, ocular surface disease index; PP, per-protocol; SD, standard deviation; TBUT, tear break-up time.

## Data Availability

In addition to summary statistics, the data points behind means, frequencies, and dispersion measures are openly available in Open Science Framework (https://osf.io) as DOI 10.17605/OSF.IO/HRPQA.

## Abbreviations

AE: Adverse events
DED: Dry eye disease
ITT: Intent-to-treat
OSDI: Ocular surface disease index
PP: Per-protocol
TBUT: Tear break-up time
BCVA: Best corrected visual acuity.
